# REEV SENSE IMUs for Spatiotemporal Gait Analysis in Post-Stroke Patients: Validation Against Optical Motion Capture

**DOI:** 10.3390/s26020667

**Published:** 2026-01-19

**Authors:** Thibault Marsan, Sacha Clauzade, Xiang Zhang, Nicolas Grandin, Tatiana Urman, Evan Linton, Samy Sibachir, Catherine E. Ricciardi, Robin Temporelli

**Affiliations:** 1 REEV SAS, 31000 Toulouse, France; sacha.clauzade@gmail.com (S.C.); nicolas.grandin.perea@pm.me (N.G.); samy.sibachir@reev.care (S.S.); robin.temporelli@reev.care (R.T.); 2 Center for Clinical and Translational Research, Massachusetts Institute of Technology, Cambridge, MA 02319, USA; xzhang88@mit.edu (X.Z.); tlevko@mit.edu (T.U.); linton@mit.edu (E.L.); c_ricci@mit.edu (C.E.R.); 3 Institute for Medical Engineering & Science, Massachusetts Institute of Technology, Cambridge, MA 02139, USA

**Keywords:** spatiotemporal parameters, wearable sensors, clinical validation, stroke survivors, assistive devices

## Abstract

**Highlights:**

**What are the main findings?**

- REEV SENSE achieves excellent reliability for temporal parameters, comparable to commercial systems, but spatial parameters degrade in slow-walking regimes (<0.4 m/s).
- Assistive device use substantially reduces accuracy for spatial parameters and swing time detection, reflecting challenges in asymmetric, low-speed gait analysis.

**What are the implications of the main finding?**

- REEV SENSE is suitable for portable, cost-effective longitudinal monitoring of temporal gait parameters in clinical post-stroke rehabilitation.
- Spatial parameters require independent validation in slow-walking conditions (<0.4 m/s); parameter-specific clinical protocols are essential for safe implementation.

**Abstract:**

Objective gait assessment is essential for post-stroke rehabilitation monitoring, yet optical motion capture systems remain inaccessible to most clinical settings due to cost and infrastructure constraints. This study assessed the validity of the REEV SENSE IMU for measuring spatiotemporal gait parameters in post-stroke individuals and evaluated assistive device effects on measurement accuracy. Twenty chronic post-stroke participants were enrolled, and fourteen completed the study (ten without an assistive device, four using a cane) after applying pre-defined exclusion criteria (walking speed <0.28 m/s, *n* = 6). Participants walked at self-selected speed while simultaneously being recorded by REEV SENSE IMUs and optical motion capture. Spatiotemporal parameters from matched heel strikes were compared using intraclass correlation coefficients (ICC), mean relative error (MRE), and Bland–Altman analysis. Temporal parameters demonstrated excellent reliability: contact time (ICC 0.96–0.99, MRE 2.77–5.45%), stride duration (ICC 0.95–0.99, MRE 2.57–2.62%), and cadence (ICC 0.98–0.99, MRE 1.80–1.93%). Spatial parameters showed greater variability, with stride length degrading substantially in slow-walking conditions (Cane group: ICC 0.76, MRE 8.60%). REEV SENSE provides reliable temporal parameter measurement comparable to commercial systems, positioning it as a practical tool for clinical gait monitoring in post-stroke rehabilitation. However, spatial parameter accuracy requires cautious interpretation in slow-walking regimes, necessitating independent validation when clinical decisions depend on precise stride length estimates.

## 1. Introduction

Gait analysis is a fundamental indicator of health status and functional mobility, making objective gait assessment essential for rehabilitation monitoring and clinical decision-making [1]. In stroke survivors, gait impairment, characterized by asymmetry, reduced walking speed, and altered spatiotemporal (SPT) parameters, directly impacts functional recovery and quality of life [2].

Accurate quantification of gait parameters is therefore critical for designing personalized rehabilitation protocols and objectively tracking treatment efficacy [3]. Traditionally, optical motion capture (OMC) systems have served as the gold standard for gait analysis, achieving high accuracy through marker-based three-dimensional tracking [4]. However, OMC systems present significant practical limitations: high cost, requirement for dedicated laboratory space, dependency on precise marker placement, and limited ecological validity due to the artificial laboratory environment [5]. These constraints restrict OMC use to specialized research centers, making objective gait assessment inaccessible to most clinical rehabilitation facilities. Wearable inertial measurement units (IMUs) offer a promising alternative, providing portable, cost-effective systems capable of continuous monitoring in real-world clinical and home-based settings [1].

Over the past decade, IMUs have emerged as a promising alternative to optical motion capture for quantifying SPT gait parameters in clinical settings. Foot-mounted IMUs, in particular, have demonstrated high validity for measuring step time, stride time, cadence, and walking speed across diverse populations. Intraclass correlation coefficients (ICC) of 0.92 for step time and 0.94 for stride time, with root mean square error (RMSE) below 40 ms, have been reported for healthy participants [6]. Brahms et al. [7] achieved ICC values of 0.89 for step length and 0.95 for stride time in healthy adults during overground running, while Küderle et al. [8] demonstrated excellent accuracy for walking speed estimation using foot-mounted IMUs compared to optical motion capture. Commercial IMU systems have also shown robust performance: Xsens MVN BIOMECH [9] achieved ICC > 0.90 for SPT parameters in clinical gait analysis with healthy participants. The APDM Mobility Lab system demonstrated good-to-excellent agreement for primary SPT parameters: gait velocity, stride length, and cadence all achieved ICC > 0.75. However, agreement was substantially lower for temporal parameters, with double support time, single support time, and swing time showing moderate to poor reliability (ICC 0.21–0.72), with particularly degraded performance in younger adults and individuals with Parkinson’s disease [10]. A comprehensive systematic review and meta-analysis synthesizing data across multiple studies confirmed consistent performance for temporal parameters (ICC = 0.85–0.98, MRE < 5–7%) in healthy and elderly populations, but revealed substantial heterogeneity in step length estimation (ICC = 0.70–0.92), with lower accuracy at slower walking speeds [11]. 

Despite the promising performance of inertial motion capture (IMC) systems in healthy populations, validation studies specifically in stroke survivors remain limited. Yang et al. [12] conducted one of the first studies applying foot-mounted IMUs to post-stroke hemiparetic gait (n = 13), utilizing acceleration and angular velocity signals to estimate walking speed and segment gait cycles. While their approach demonstrated good performance for walking speed estimation, the study was limited in scope and did not comprehensively validate all SPT parameters. More recently, Lefeber et al. [13] validated foot-mounted IMU sensors against optical motion capture in stroke patients (n = 20), demonstrating good validity for step time, stride time, and cadence, with excellent reproducibility for temporal parameters. However, this study did not systematically examine the influence of assistive device use on measurement accuracy. Lanotte et al. [14] extended this work by investigating how walking speed and gait asymmetry affect IMC accuracy in post-stroke populations, revealing that measurement performance is substantially influenced by these factors. Most recently, Desai et al. [15] provided a comprehensive validation of commercial IMC systems in post-stroke individuals, confirming good-to-excellent validity for primary SPT parameters. Collectively, these studies suggest that IMC systems can provide valid measurements in stroke populations, yet critical gaps remain regarding the standardization of synchronization protocols, the systematic evaluation of assistive device effects, and the characterization of performance across varying levels of gait impairment severity.

Several important limitations characterize the existing literature on IMU-based gait analysis that this study aims to address. First, synchronization protocols between IMU and optical motion capture systems are rarely standardized or explicitly reported across studies, introducing potential systematic errors and limiting reproducibility of findings [16]. Second, the existing literature on IMU-based gait analysis in stroke populations remains sparse, with most validation studies conducted in healthy or mildly impaired cohorts. This limits the generalizability of performance metrics established in healthy populations to severely impaired stroke gait patterns, particularly regarding the reliability of asymmetry and variability measures [11]. Third, few studies explicitly distinguish or control for the level of walking assistance (e.g., use of canes, walkers, or therapist support) during gait assessment, making it difficult to isolate the contribution of IMU measurement error from confounding factors related to assistive device use or external support.

This study addresses these gaps by providing rigorous validation of foot-mounted IMU sensors for SPT gait analysis in stroke patients in controlled clinical conditions, with explicit attention to synchronization methodology and assistive device documentation. Specifically, the REEV SENSE IMU system (REEV SAS, Labège, France), approved by the FDA as a class I medical device, was developed to objectively assess rehabilitation outcomes in clinical populations using wearable motion analysis sensors. A key feature of REEV SENSE is its streamlined computational architecture: SPT parameters are calculated directly within the IMU hardware without relying on magnetometer data, and results are transmitted to the application within one minute. This design enables rapid feedback and reduces computational burden on external devices. The system measures gait parameters non-invasively, with sensors attached to the patient’s shoe. When paired with the REEV SENSE application, the system provides a tool for gait analysis in clinical settings, allowing physical therapists to obtain measurements with minimal setup time.

Evaluating sensor performance with assistive devices is crucial, as these tools substantially modify gait mechanics and introduce variability in sensor signal characteristics. Most post-stroke patients rely on canes, walkers, or orthoses during ambulation, yet the effects on IMU measurement accuracy have not been systematically studied in the literature. Given the asymmetric and altered movement patterns characteristic of stroke survivors, robust and sensitive measurement tools are needed to quantify subtle gait impairments and reliably monitor rehabilitation outcomes over time. Objective gait monitoring directly supports clinical decision-making and enables personalized rehabilitation planning tailored to individual patient needs.

This study addresses three specific gaps in the existing literature. First, while prior validation studies have examined IMU systems in post-stroke populations, none have systematically characterized the effects of assistive device use (canes, walkers) on measurement accuracy, a critical factor in clinical rehabilitation where these devices are routinely employed. Second, synchronization protocols between IMU and optical motion capture systems are rarely standardized or explicitly reported, limiting reproducibility. We implement a rigorous cross-correlation-based approach with transparent methodology. Third, most prior validation studies focus on healthy or mildly impaired gait at faster speeds; this study validates performance in clinically relevant slow-walking regimes (<0.4 m/s) characteristic of household ambulation in post-stroke populations.

Therefore, the primary objective of this study was to assess the concurrent validity of the REEV SENSE IMU system compared to an OMC reference standard for measuring SPT gait parameters in post-stroke individuals. A secondary objective was to evaluate whether the accuracy of IMC-derived SPT measurements is influenced by the use of assistive devices (canes, walkers) commonly employed by stroke survivors during gait. We hypothesized that REEV SENSE IMUs would provide accurate estimations of SPT parameters during gait, with mean relative error (MRE) remaining below 5%. We further hypothesized that assistive device use would introduce measurable differences in IMC accuracy, particularly in post-stroke individuals with asymmetric gait patterns.

Such validation would support the clinical adoption of REEV SENSE for objective, real-time gait assessment in rehabilitation settings, enabling evidence-based monitoring of stroke recovery and improving accessibility to objective gait analysis for stroke survivors.

## 2. Materials and Methods

### 2.1. Participants

Twenty chronic post-stroke individuals (>6 months post-stroke) were enrolled in this study (Table 1). All participants were over 18 years old and capable of walking independently or with assistance. Participants were stratified by their personal assistive device use: ten used no device (None group), eight used canes (Cane group), and two used walkers (Walker group). Each participant maintained consistent device use throughout all trials.

### 2.2. Protocol

Each participant was asked to walk at self-selected speed along a 10 m walkway and complete up to three round trips (maximum distance: 60 m per participant). Participants used their own assistive device (cane, walker, or none) consistently throughout all trials. Rest breaks were provided as needed between trials. The entire protocol lasted approximately one hour per participant, including sensor setup and calibration time.

### 2.3. Exclusion Criteria

Participants with walking speed below 0.28 m/s were excluded, as this threshold represents the lower boundary of unlimited household ambulation in stroke populations [17], below which gait patterns become highly variable and unreliable for validation purposes. Participants with fewer than 4 common steps between IMC and OMC systems were excluded, as a minimum of 4 footsteps is required to obtain reliable ICC estimates (ICC > 0.90) for spatiotemporal parameters [18].

### 2.4. Consent

Participants were recruited through Clinical Connection and an online platform, with eligibility screening performed by CCTR staff at MIT. This study is part of the REEV SENSE project investigating gait analysis in post-stroke populations (SENS-AG, NCT06234878) and was approved by the Institutional Review Board (IRB Protocol #20234678). Eligible participants received the informed consent form via email prior to enrollment discussions. The consent process was conducted remotely via phone or video conference, allowing participants adequate time to ask questions and make an informed decision about their participation. Electronic consent was obtained using REDCap, a HIPAA-compliant research data capture system, with signed copies provided to participants via email or physical mail. Participant identification was verified at the time of consent and again at the study visit to ensure proper documentation and study protocol compliance.

### 2.5. Instrumentation

Simultaneous data collection was performed using optical motion capture (OMC) and inertial measurement units (IMC). The OMC system comprised twelve infrared cameras (Qualisys, Göteborg, Sweden) operating at 100 Hz, capturing participant movements using 28 reflective markers corresponding to a lower body Common Gait Model 2.3 marker set [19]. For spatiotemporal analysis, only pelvis and foot markers were utilized.

The IMC system consisted of two REEV SENSE IMUs (REEV SAS, Labège, France) based on the LSM6DSL sensor (STMicroelectroncics, Genève, Switzerland). Sensors recorded 3D linear acceleration (±16 g, sensitivity 0.488 × 10^−3^ g, with g = 9.81 m·s^2^) and 3D angular velocity (±2000°/s, sensitivity 0.07°/s) at a 100 Hz sampling frequency. The REEV SENSE IMUs did not include a magnetometer to avoid errors caused by magnetic disturbances common in clinical environments. All specifications are based on manufacturer data.

The REEV SENSE IMUs did not include a magnetometer in order to avoid errors caused by magnetic disturbances, which are common in clinical environments and can significantly affect orientation estimates in traditional IMU systems.

**Figure 1:**
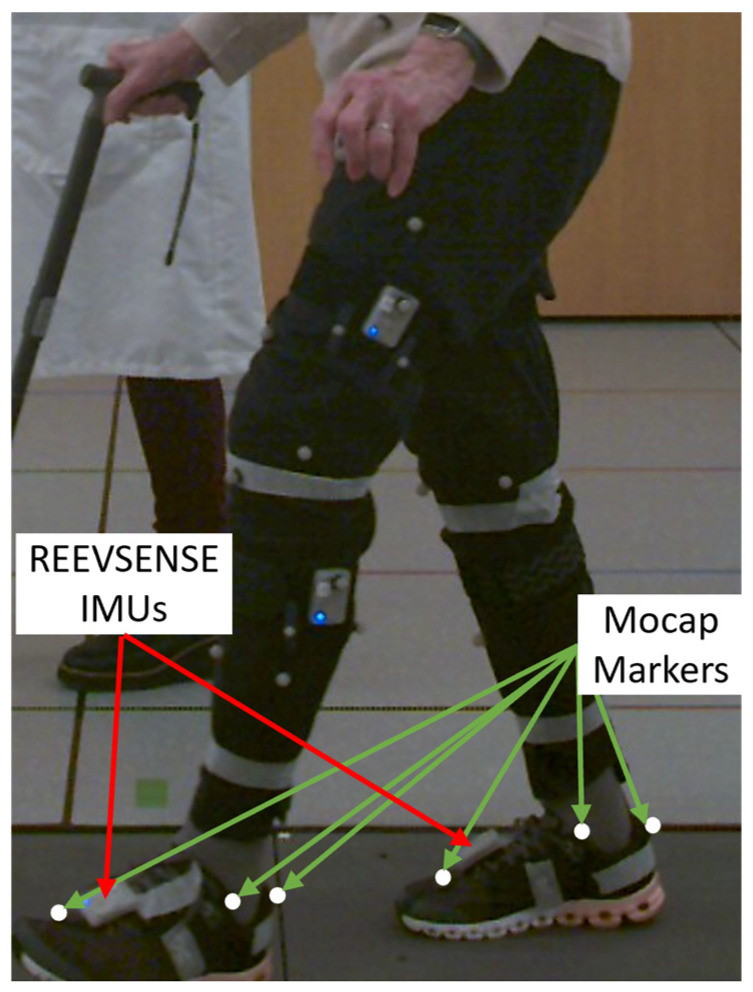
Example of IMU and marker placement on a participant for spatiotemporal parameter analysis. Only foot markers/sensors are indicated (green arrows for optical motion capture markers, red arrows for REEV SENSE IMUs). Placement was symmetrical on both lower limbs.

Once the subject was equipped with the reflective markers, the REEV SENSE IMUs were placed on the participants (Figure 1). The IMUs on the shoes were attached using magnetic plates placed between the shoelaces. The IMC system communicated with a computer running the REEV SENSE PC application via a Bluetooth protocol. Data were recorded at 100 Hz on the PC application and stored locally for offline processing. Both sensors were placed at once and connected to the REEV SENSE PC application, which enabled simultaneous recording from both sensors.

### 2.6. Data Processing

**Figure 2:**
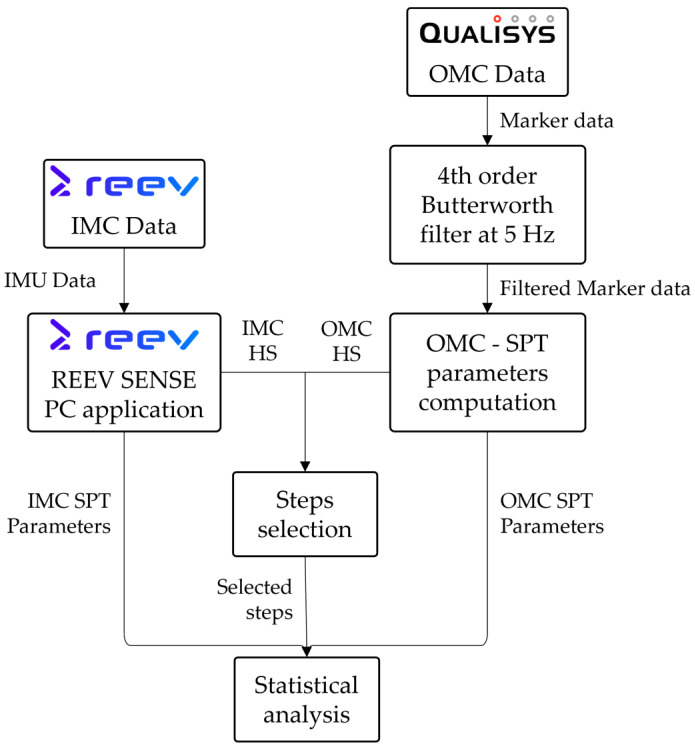
Data processing workflow for spatiotemporal parameter extraction and synchronization.

The data processing workflow is illustrated in Figure 2. Raw IMU and OMC data were processed in parallel to extract spatiotemporal parameters. Cross-correlation-based synchronization temporally aligned both systems. Matched heel strikes were identified, and corresponding spatiotemporal parameters were extracted for validation analysis.

#### 2.6.1. IMC–SPT Parameters Computation

Raw IMU data were processed by the REEV SENSE application using proprietary algorithms embedded in the IMU hardware, without relying on magnetometer data. SPT parameters were computed directly during data acquisition, with results subsequently displayed on the PC application. Temporal parameters include contact time, swing time, stride time, double support time (all in seconds), and cadence (strides per minute) for both paretic and non-paretic limbs. Spatial parameters include stride length (meters) and gait velocity (meters per second). The HS and TO were also computed by the REEV SENSE IMUs using proprietary algorithms operating on acceleration and angular velocity data.

#### 2.6.2. OMC–SPT Parameters Computation

Regarding the OMC, marker data were filtered using a 4th order Butterworth low-pass filter with a cutoff frequency of 5 Hz to remove high-frequency noise. Heel strikes and toe-offs were identified from the OMC data using the relative distance between sacral and foot markers (RDSF) method. This approach was selected based on its demonstrated accuracy, even on pathological gait, compared to the gold standard: force platforms [20].

Spatiotemporal parameters were computed from the identified gait events as follows: Stride time was calculated as the time interval between two consecutive heel strikes on the same foot. Cadence was derived as the number of strides per minute (60/strides time). Contact time was defined as the duration between successive heel strike and toe-off events. Swing time was calculated as the time between toe-off and the subsequent heel strike. Stride length was computed as the anterior–posterior displacement of the foot marker between consecutive heel strikes. Gait velocity was derived as the ratio of stride length to stride time. Double support time was calculated as the sum of contact times for both limbs minus the stride duration, representing the period when both feet are in contact with the ground simultaneously. All temporal parameters were expressed in seconds, spatial parameters in meters, and velocity in meters per second. These parameters were computed using this methodology to match the calculation approach implemented in the REEV SENSE IMU system, ensuring direct comparability between the two measurement systems.

#### 2.6.3. Synchronization of OMC and IMC

The synchronization was performed offline prior to heel strike matching. To ensure accurate temporal alignment between IMU and OMC data, a cross-correlation-based synchronization method was implemented. For the IMU system, the raw vertical acceleration from the foot-mounted sensor was extracted and expressed in the ground reference frame (vertical axis). For the OMC system, foot position was computed as the average position of markers placed on the heel and toe clusters, which was then differentiated twice to obtain vertical acceleration.

Cross-correlation analysis was performed between the IMU vertical acceleration and the OMC-derived vertical acceleration to identify the optimal time lag between the two signals. The time lag corresponding to the maximum cross-correlation coefficient was applied to temporally align all IMU and OMC data prior to further analysis. This approach ensures that gait events (heel strikes and toe-offs) detected by both systems are synchronized to the same temporal reference frame, eliminating potential systematic timing errors that could bias validation results.

#### 2.6.4. Heel Strike Matching

Heel strikes detected by both systems were matched using a one-to-one matching algorithm with a temporal tolerance of 10 frames (so 0.1 s at 100 Hz). For each OMC heel strike, the closest IMU heel strike within the tolerance window was identified. Only heel strikes with temporal differences <100 ms were retained as matched steps to ensure robust temporal alignment between systems and minimize synchronization artifacts [21]. This tolerance threshold is consistent with the inherent detection variability of gait event algorithms (±20–50 ms) and accounts for inter-system latency. Spatiotemporal parameters corresponding to matched heel strikes were extracted from both systems for statistical analysis.

### 2.7. Statistical Analysis

For each spatiotemporal parameter, differences between ReevSense and Mocap measurements were computed as (IMC value − OMC Value). Outliers beyond ±1.96 SD from the mean difference were excluded prior to final analysis to ensure robust statistical estimates and remove measurement artifacts. This threshold corresponds to the 95% confidence interval and aligns with standard Bland–Altman methodology [22].

Validation metrics were selected following a systematic approach to benchmarking IMU and optical motion capture systems. Following [23], metrics from three primary categories—correlation coefficients, Bland–Altman analyses, and error metrics—were employed.

For each spatiotemporal parameter, the following metrics were computed: intraclass correlation coefficient (ICC (3,1)) for two-way mixed-effects model with absolute agreement, mean bias (Bias %) and limits of agreement (LoA %) following Bland–Altman methodology (expressed as percentage of OMC reference values), and mean relative error (MRE %). Normality of differences was assessed using the Shapiro–Wilk test for each parameter. For normally distributed differences (*p* > 0.05), paired t-tests were performed and for non-normally distributed differences, Wilcoxon signed-rank tests were used. This adaptive approach ensures appropriate statistical testing based on data characteristics and is particularly suitable for small sample sizes. Statistical significance was set at α = 0.05.

All analyses were conducted in Python 3.12 (Python Software Foundation, https://www.python.org) using NumPy 1.26.4 (https://numpy.org), SciPy 1.13.1 (https://scipy.org), and Pandas 2.3.3 (https://pandas.pydata.org) libraries.

## 3. Results

### 3.1. Participant Characteristics and Data Exclusion

Of the twenty initial participants, six were excluded based on exclusion criteria (Section 2.3): the two participants using a walker were excluded due to a walking speed below 0.28 m/s, and four participants from the Cane group were excluded (two due to a walking speed <0.28 m/s, and two due both to a walking speed <0.28 m/s and fewer than four matched steps between systems). The final cohort consisted of 14 participants: 10 in the None group and 4 in the Cane group. Updated participant demographics are presented in Table 2.

### 3.2. Mean Comparison Summary

Mean values and standard deviations for all spatiotemporal parameters measured by both IMC and OMC systems are presented in Table 3, stratified by assistive device group (None, Cane) and combined cohort (All).

Contact time ranged from 0.82 ± 0.15 s (paretic side, None group, IMC) to 1.25 ± 0.20 s (non-paretic side, Cane group, IMC). Swing time values ranged from 0.36 ± 0.04 s (non-paretic side, None group, IMC) to 0.48 ± 0.07 s (paretic side, Cane group, OMC).

Stride time increased with assistive device use, ranging from 1.29 ± 0.23 s (None group, IMC) to 1.69 ± 0.17 s (Cane group, OMC). Double support time similarly increased with cane use, from 0.45 ± 0.13 s (None group, IMC) to 0.80 ± 0.16 s (Cane group, IMC).

Cadence decreased with assistive device use from 47.38 ± 8.15 strides/min (None group, IMC) to 35.86 ± 4.12 strides/min (Cane group, IMC). Stride length was substantially reduced in the Cane group (0.62 ± 0.08 m, IMC; 0.66 ± 0.10 m, OMC) compared to the None group (0.99 ± 0.13 m, IMC; 1.03 ± 0.14 m, OMC). Stride speed showed the largest differences between groups, ranging from 0.37 ± 0.10 m/s (Cane group, IMC) to 0.82 ± 0.21 m/s (None group, OMC).

Overall, IMC and OMC measurements showed similar patterns across all parameters and groups, with minimal differences in mean values between systems.

### 3.3. Validation Metrics Summary

Validation metrics for all spatiotemporal parameters are presented in Table 4, with detailed Bland–Altman plots in Supplementary Figure S1 and ICC heatmap in Supplementary Figure S2.

Temporal parameters demonstrated excellent reliability. Contact time showed ICC 0.96–0.99 (MRE 2.77–5.45%, LoA 7.55–10.26%), with minimal bias (0.04–4.18%). Swing time exhibited lower performance (ICC 0.79–0.95, MRE 4.72–8.70%, LoA 11.15–18.00%), with higher bias on the paretic side in the None group (6.19%). Stride duration and cadence were highly reliable (ICC 0.95–0.99, MRE 1.80–2.62%), with narrow limits of agreement (4.57–6.67%). Double support time showed moderate reliability (ICC 0.94–0.96, MRE 6.53–8.39%) with the widest limits of agreement (13.77–18.93%).

Spatial parameters were more variable. Stride length ranged from poor-to-excellent (ICC 0.76–0.97, MRE 4.78–8.60%), with substantially degraded accuracy in the Cane group (ICC 0.76, MRE 8.60%). Stride speed showed excellent ICC (0.90–0.99) but variable MRE (4.72–10.99%), with poor performance in slow-walking conditions (MRE 10.99%).

Systematic bias was statistically significant for most parameters (*p* < 0.001), except contact time on the paretic side in the Cane group (*p* = 0.786) and double support time across the combined cohort (*p* = 0.231).

## 4. Discussion

The spatiotemporal parameters measured in this study align with post-stroke gait patterns documented in the literature. Walking speed ranged from 0.37 to 0.80 m/s, stride length from 0.62 to 1.03 m, and cadence from 35 to 47 strides/min, all within the expected ranges for chronic stroke survivors [24]. Speed-matched comparisons with healthy controls revealed characteristic asymmetries in temporal parameters, including prolonged swing time on the paretic side and increased double support time [25], consistent with known post-stroke gait alterations. The use of a cane resulted in reduced walking speed and stride length with increased double support time, reflecting typical compensatory strategies in household ambulation. These findings validate the representativeness of our sample and support the use of spatiotemporal parameters as sensitive indicators of gait abnormality in stroke survivors [26].

Our temporal parameters demonstrated excellent reliability: contact time (ICC > 0.96, MRE < 5.45%); stride duration and cadence (ICC > 0.95, MRE < 2.62%). Swing time showed lower performance (ICC 0.79–0.95, MRE 4.72–8.70%), likely due to challenges in detecting toe-off from foot-mounted sensors. Spatial parameters were more variable: stride length ranged from poor to excellent (ICC 0.76–0.97, MRE 4.78–8.60%), with degraded accuracy in the Cane group (ICC 0.76, MRE 8.60%), reflecting the effects of slow speed and asymmetry on double-integration-based position estimation. Stride speed showed excellent ICC but variable MRE (4.72–10.99%), with poor performance in the Cane group (MRE 10.99%) due to accumulated spatial errors. Overall, the system reliably measured temporal parameters but faced challenges with spatial parameters and swing phase detection in slow, asymmetric walking.

This study tested two primary hypotheses regarding REEV SENSE performance in post-stroke gait assessment. The first hypothesis, that REEV SENSE IMUs would provide accurate estimations of spatiotemporal parameters with mean relative errors remaining below 5%, was partially supported. Temporal parameters (contact time, stride duration, cadence) consistently achieved an MRE < 5% across all groups and limbs, meeting the predefined accuracy threshold and validating the system’s reliability for these parameters. However, spatial parameters showed heterogeneous results: stride length in the None group achieved an MRE < 5% (4.78–5.38%), but degraded substantially in the Cane group (MRE 8.60%), failing to meet the 5% threshold in slow-walking conditions (<0.4 m/s which corresponds to household ambulator speed [17]). This pattern suggests that the 5% accuracy criterion is achievable for temporal parameters across diverse walking conditions but remains challenging for spatial parameters during asymmetric, slow-speed gait, a critical distinction for clinical implementation. The second hypothesis, that assistive device use would introduce measurable differences in IMC accuracy, was strongly supported. Cane use resulted in substantially degraded performance across spatial parameters (stride length ICC 0.76 vs. 0.97; stride speed MRE 10.99% vs. 4.72%) and increased variability in swing time detection (MRE 8.70% vs. 4.72%), reflecting the compounding effects of reduced walking speed, increased gait asymmetry, and altered movement patterns characteristic of cane-assisted ambulation. These findings underscore the importance of assistive device-specific validation protocols and highlight the need for context-dependent accuracy thresholds in clinical gait assessment.

Our validation results demonstrate performance comparable to commercial IMU systems, particularly Xsens MVN, which is the most widely used system in clinical settings. For spatiotemporal parameters in healthy gait, Xsens reports RMSE values of 2.61–3.52 cm for step/stride length and biases not centered around zero of −1.37 to −2.47 cm [16]. Our system achieved similar accuracy with MRE < 5% for stride duration, contact time, and cadence in the None group, indicating equivalent performance for temporal parameters. However, our stride length showed higher variability (MRE > 5% in the Cane group), consistent with Xsens’ reported limitations in spatial parameter estimation during asymmetric gait (step width RMSE = 6.53–6.82 cm, bias = −4.29 to −4.67 cm).

Beyond commercial systems, findings align with broader IMU validation literature across diverse populations. The APDM Opal IMUs were validated for 39 healthy young adults [27], and they reported excellent validity for stride time (ICC > 0.90) and good-to-excellent validity for stride length and swing/stance time. We demonstrated comparable or superior performance for temporal parameters (stride duration, contact time, and cadence, all with ICC > 0.95 in the None group), although stride length showed greater variability in asymmetric gait, consistent with APDM’s reported limitations. In post-stroke populations specifically, the APDM Opal IMUs were validated with 23 chronic stroke survivors [15] and reported fair-to-excellent agreement for temporal parameters (CCC 0.56–0.98) and excellent agreement for spatial parameters (CCC > 0.90), with excellent test–retest reliability (ICC > 0.90). Our MRE thresholds for temporal parameters (<5%) exceeded these benchmarks, suggesting higher accuracy, though direct comparison is limited by differences in populations (chronic vs. mixed acute/subacute stroke) and walking conditions (treadmill vs. overground). Meta-analytic evidence [11] synthesizing 82 studies confirms that temporal parameters (step and stride times) demonstrate excellent validity across IMU systems, while stride length shows more variable results, a pattern directly reflected in the present data. This suggests temporal parameter estimation is a robust feature of IMU-based gait analysis, whereas spatial parameters remain a challenge, particularly during asymmetric walking patterns.

Despite these encouraging findings, several limitations warrant consideration. The sample size (n = 14 post-stroke participants after exclusions) is modest, limiting statistical power and generalizability, particularly for subgroup comparisons (None vs. Cane cohorts). However, ICC values > 0.95 for temporal parameters indicate excellent reliability even with small samples, and consistency of results across subgroups suggests robust underlying patterns rather than statistical artifacts. Exclusion criteria were pre-defined and methodologically justified: participants with walking speed < 0.28 m/s were excluded because gait patterns become highly variable below this threshold, and participants with fewer than four matched steps were excluded because ICC estimation requires minimum step counts. We acknowledge the sample size as a limitation and recommend future validation in larger, more diverse post-stroke populations. Future studies should incorporate validated clinical scales and Passing–Bablok regression analysis for enhanced statistical rigor and generalizability.

While assistive device use and walking speed provide indirect information on impairment severity, the absence of standardized motor function scores (e.g., Fugl-Meyer assessment) represents a limitation of this study. Future work should incorporate validated clinical scales to enable clearer stratification of impairment severity.

Population specificity presents both a limitation and a strength: the predominantly elderly post-stroke cohort restricts direct applicability to younger or other neurological populations, yet provides clinically relevant evidence for the target rehabilitation population. Future work should systematically explore whether findings generalize across age groups and pathologies.

A critical technical constraint emerged: the system demonstrated reduced accuracy in slow-walking regimes (Cane group, mean speed < 0.5 m/s), precisely the condition most relevant to post-stroke rehabilitation. This reflects a known challenge in IMU-based gait analysis, as larger errors in spatiotemporal parameters have been documented for slow walking speeds [28] and stems partly from laboratory constraints (multiple turning points, limited usable steps per trial, residual outliers despite exclusion). While this limitation highlights the need for algorithm refinement in pathological, low-speed conditions, the acceptable performance achieved even under these challenging conditions suggests the system’s potential with targeted improvements.

REEV SENSE demonstrates high reliability for temporal parameters (ICC > 0.95, MRE < 2.62%), positioning it as a practical tool for longitudinal gait monitoring in post-stroke rehabilitation. Performance is comparable to commercial systems (Xsens MVN, APDM Opal) while offering distinct clinical advantages. Unlike optical motion capture systems requiring dedicated laboratory infrastructure and marker placement expertise, REEV SENSE enables objective gait assessment in clinic-based settings with minimal setup time. Rapid feedback (<1 min) supports real-time clinical decision-making during rehabilitation sessions, enabling clinicians to adjust therapeutic strategies based on objective metrics rather than subjective observation. Temporal parameters (contact time, stride duration, cadence) are sensitive indicators of motor recovery and respond to rehabilitation interventions, enabling longitudinal tracking of rehabilitation progress and evidence-based adjustment of treatment protocols. However, degraded spatial parameter accuracy in slow-walking regimes (<0.5 m/s, MRE 8.60% in Cane group) necessitates cautious interpretation in household ambulation contexts. REEV SENSE occupies a complementary opportunity: continuous, portable monitoring of temporal gait characteristics in clinical settings, with optical motion capture reserved for high-precision spatial analysis or validation when clinical decisions depend on stride length estimates.

Temporal parameter reliability supports tracking post-stroke gait recovery, as contact time, stride duration, and cadence are sensitive indicators of motor recovery and respond to rehabilitation interventions. Serial measurements enable clinicians to quantify recovery trajectories and adjust therapeutic strategies accordingly. Real-time feedback capabilities could enhance motor learning and patient engagement during rehabilitation sessions.

Safe clinical integration of REEV SENSE requires parameter-specific protocols: temporal parameters should serve as primary outcome measures, while spatial parameters should be treated as supplementary information requiring independent validation when clinical decisions depend on precise stride length estimates. Establishing minimum walking speed thresholds (>0.5 m/s) below which spatial estimates should not be used without validation is essential. Periodic validation against optical motion capture or instrumented walkways is recommended, particularly when clinical decisions depend on spatial parameters or when patient gait patterns change substantially. Standardized sensor placement protocols and regular calibration checks minimize systematic errors. When reporting REEV SENSE-derived metrics in clinical documentation, clinicians should clearly distinguish high-confidence temporal parameters from lower-confidence spatial estimates, with explicit notation of measurement uncertainty.

To enhance clinical utility, REEV SENSE development should prioritize algorithm optimization for slow-speed gait through speed-adaptive or machine learning approaches trained on pathological walking data. Swing phase detection refinement, developing improved toe-off detection algorithms, would enhance asymmetry metrics critical for post-stroke assessment. Validation should extend to diverse post-stroke populations with establishment of population-specific accuracy profiles and minimal detection change thresholds. Integration of clinical decision support software that automatically flags unreliable measurements and suggests when independent validation is warranted would facilitate safe clinical adoption. Maintaining comparative validation against emerging IMU systems ensures continued clinical relevance as technology evolves.

## 5. Conclusions

REEV SENSE demonstrates high accuracy for temporal parameter monitoring in post-stroke gait assessment. The system’s portability and performance are comparable to commercial platforms, enabling longitudinal tracking of gait parameters in clinical settings. However, acknowledged limitations in spatial parameter estimation during slow (<0.4 m/s), asymmetric walking require careful protocol implementation and clinician awareness. With targeted algorithm refinement and validation in diverse populations, IMU-based systems, including REEV SENSE, enable quantitative gait analysis in clinical settings without requiring laboratory infrastructure.

## Figures and Tables

**Table 1:** Initial participants demographics, presented as mean ± standard deviation.

Assist.	N	Height (m)	Age (y. o.)	Weight (kg)
None	10	1.74 ± 0.09	55.20 ± 9.341	76.43 ± 9.704
Cane	8	1.67 ± 0.10	54.80 ± 13.19	74.78 ± 18.86
Walker	2	1.80 ± 0.22	54.78 ± 9.191	96.16 ± 24.70
All	20	1.71 ± 0.11	55.44 ± 11.61	75.75 ± 16.73

**Table 2:** Final participants demographics, presented as mean ± standard deviation.

Assist.	N	Height (m)	Age (y.o.)	Weight (kg)
None	10	1.74 ± 0.08	57.20 ± 9.34	76.43 ± 9.69
Cane	4	1.68 ± 0.12	71.00 ± 14.09	79.03 ± 24.64
All	14	1.72 ± 0.09	61.14 ± 12.17	77.17 ± 14.38

**Table 3:** Descriptive statistics of spatiotemporal parameters measured by IMC and OMC across assistive device groups (mean ± SD). IMC: inertial measurement unit system; OMC: optical motion capture; None: no assistive device; Cane: assisted by a cane; All: combined cohort.

Parameter	Side	None		Cane		All	
		IMC	OMC	IMC	OMC	IMC	OMC
Contact time (s)	Non-paretic	0.95 ± 0.24	0.95 ± 0.24	1.25 ± 0.20	1.23 ± 0.19	1.06 ± 0.27	1.05 ± 0.26
	Paretic	0.82 ± 0.15	0.85 ± 0.16	1.20 ± 0.21	1.20 ± 0.18	0.98 ± 0.26	1.00 ± 0.24
Swing time (s)	Non-Paretic	0.36 ± 0.04	0.37 ± 0.03	0.44 ± 0.10	0.45 ± 0.07	0.38 ± 0.07	0.40 ± 0.06
	Paretic	0.47 ± 0.10	0.44 ± 0.09	0.46 ± 0.1	0.48 ± 0.07	0.47 ± 0.10	0.46 ± 0.09
Stride time (s)		1.29 ± 0.23	1.30 ± 0.23	1.68 ± 0.18	1.69 ± 0.17	1.44 ± 0.28	1.45 ± 0.28
Double support time (s)		0.45 ± 0.13	0.48 ± 0.13	0.80 ± 0.16	0.76 ± 0.15	0.58 ± 0.22	0.58 ± 0.20
Cadence (stride/min)		47.38 ± 8.15	47.21 ± 8.04	35.86 ± 4.12	35.64 ± 3.93	42.9 ± 8.88	42.71 ± 8.81
Stride length (m)		0.99 ± 0.13	1.03 ± 0.14	0.62 ± 0.08	0.66 ± 0.10	0.86 ± 0.21	0.90 ± 0.22
Stride speed (m·s^−1^)		0.80 ± 0.21	0.82 ± 0.21	0.37 ± 0.10	0.40 ± 0.12	0.65 ± 0.27	0.67 ± 0.27

**Table 4:** Validation metrics for spatiotemporal parameters comparing IMC and OMC. Status: **✓** Excellent (ICC > 0.95, MRE < 5%); ◆ Acceptable (ICC > 0.85, MRE < 10%); **✗** Poor (ICC < 0.85 or MRE > 10%). LoA: limits of agreement; MRE: mean relative error; ICC: intraclass correlation. n: number of steps. * Significant difference between methods (*p* < 0.05).

Parameter	Side	Group	Bias (%)	LoA (%)	MRE (%)	ICC	*p*-Value	Status	n
**Contact ** ** time ** ** (s)**	**Non ** ** paretic**	None	0.56	7.55	2.77	0.99	0.086	**✓**	232
		Cane	1.41	9.23	4.12	0.96	<0.001 *	**✓**	133
		All	1.02	8.40	3.29	0.99	<0.001 *	**✓**	365
	**Paretic**	None	4.18	10.26	5.45	0.96	<0.001 *	**◆**	210
		Cane	0.04	8.54	3.79	0.96	0.786	**✓**	150
		All	2.14	10.18	4.83	0.98	<0.001 *	**✓**	360
**Swing ** ** time ** ** (s)**	**Non paretic**	None	2.74	11.15	4.72	0.79	<0.001 *	**◆**	222
		Cane	2.92	17.57	8.70	0.89	<0.001 *	**◆**	118
		All	3.15	13.79	6.03	0.90	<0.001 *	**◆**	336
	**Paretic**	None	6.19	13.10	7.05	0.95	<0.001 *	**◆**	218
		Cane	3.03	18.00	8.62	0.88	<0.001 *	**◆**	155
		All	2.50	17.33	7.54	0.91	<0.001 *	**◆**	373
**Stride duration ** ** (s)**		None	0.45	6.67	2.62	0.98	0.060	**✓**	204
		Cane	0.54	6.57	2.59	0.95	0.031 *	**✓**	126
		All	0.52	6.51	2.57	0.99	<0.001 *	**✓**	330
**Cadence ** ** (strides/min)**		None	0.36	4.64	1.93	0.99	0.002 *	**✓**	212
		Cane	0.64	4.57	1.80	0.98	<0.001 *	**✓**	128
		All	0.44	4.74	1.93	0.99	<0.001 *	**✓**	340
**Double support ** ** (s)**		None	4.74	18.93	8.39	0.94	<0.001 *	**◆**	173
		Cane	4.39	13.77	6.53	0.94	<0.001 *	**◆**	108
		All	0.67	18.72	7.83	0.96	0.231	**◆**	281
**Stride length ** ** (m)**		None	3.41	9.54	4.78	0.93	<0.001 *	**✓**	196
		Cane	5.38	18.57	8.60	0.76	<0.001 *	**◆**	106
		All	3.92	11.95	6.13	0.97	<0.001 *	**✓**	302
**Stride speed ** ** (m·s−1)**		None	2.37	9.92	4.72	0.98	<0.001 *	**✓**	196
		Cane	5.73	23.41	10.99	0.90	<0.001 *	**✗**	114
		All	3.05	12.79	6.96	0.99	<0.001 *	**✓**	310

## Data Availability

Data are unavailable due to privacy restrictions.

## Supplementary Materials

The following supporting information can be downloaded at https://www.mdpi.com/article/10.3390/s26020667/s1, Supplementary Figure S1: Bland-Altman plots for all spatiotemporal parameters comparing IMC and OMC. Each plot shows data points colored by assistive device group (None in blue, Cane in orange). Bias and limits of agreement (LoA) are calculated for the combined cohort (All).; Supplementary Figure S2: Intraclass correlation coefficient (ICC(3,1)) heatmap for spatiotemporal parameters comparing IMC and OMC across assistive device groups (None, Cane). ICC values range from 0.75 (red) to 1.0 (green), with higher values indicating better agreement between systems. ICC(3,1) represents a two-way mixed effects model with absolute agreement.

## Abbreviations

The following abbreviations are used in this manuscript:

OMC	Optical Motion Capture
IMU	Inertial Measurement Unit
IMC	Inertial Motion Capture
ICC	Intraclass Correlation
RMSE	Root Mean Square Error
SPT	Spatiotemporal
LoA	Limits of Agreement
MRE	Mean Relative Error
