# Supplementary material for: REEV SENSE IMUs for Spatiotemporal Gait Analysis in Post-Stroke Patients: Validation Against Optical Motion Capture
Source: Sensors (Basel). 2026 Jan 19;26(2):667. doi: 10.3390/s26020667 (PMC12845817; doi:10.3390/s26020667)
Supplement: Supplementary file 1 [file sensors-26-00667-s001.zip › sensors-4033885-supplementary.pdf]

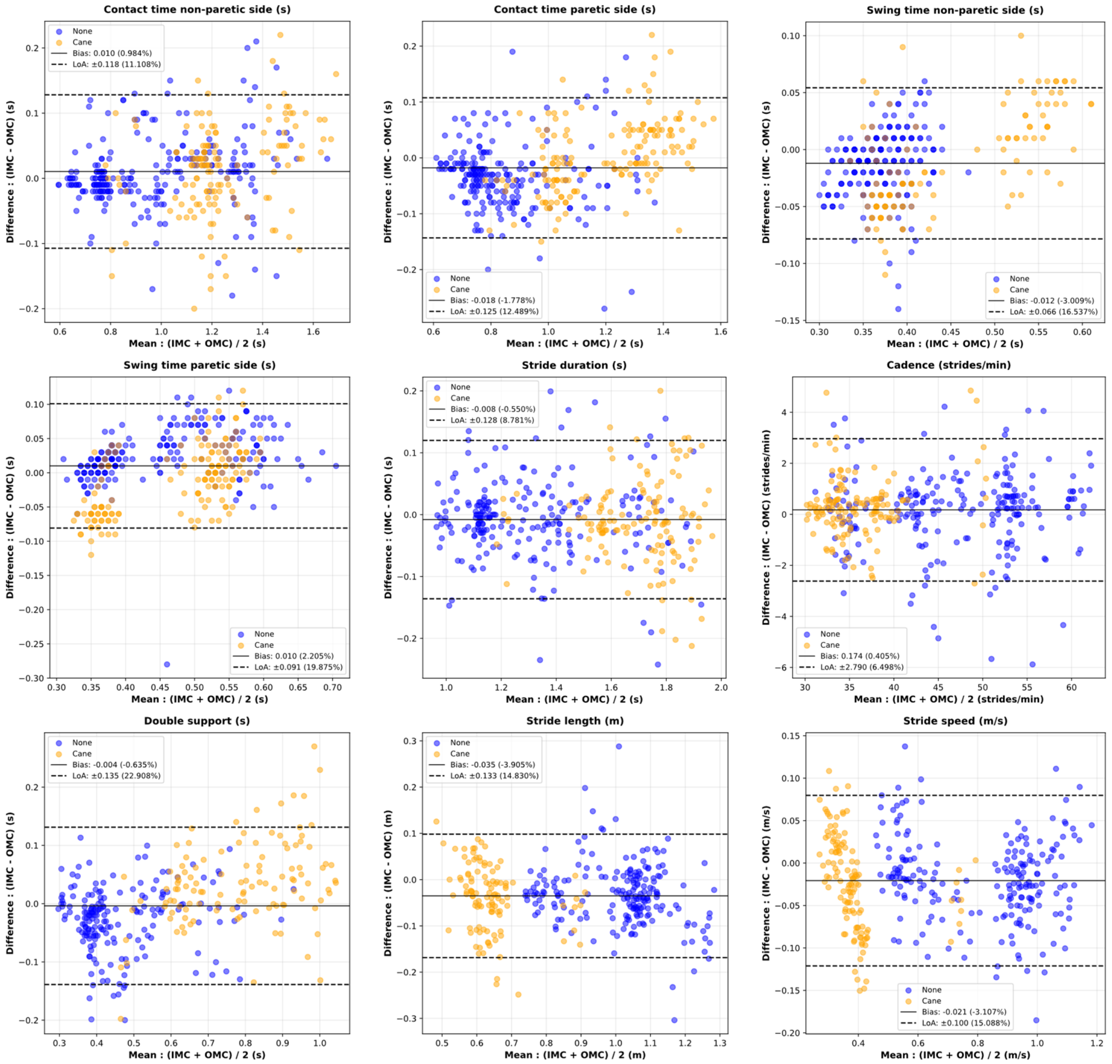

Supplementary Figure S1. Bland-Altman plots for all spatiotemporal parameters comparing IMC and OMC. Each plot shows data points colored by assistive device group (None in blue, Cane in orange). Bias and limits of agreement (LoA) are calculated for the combined cohort (All).

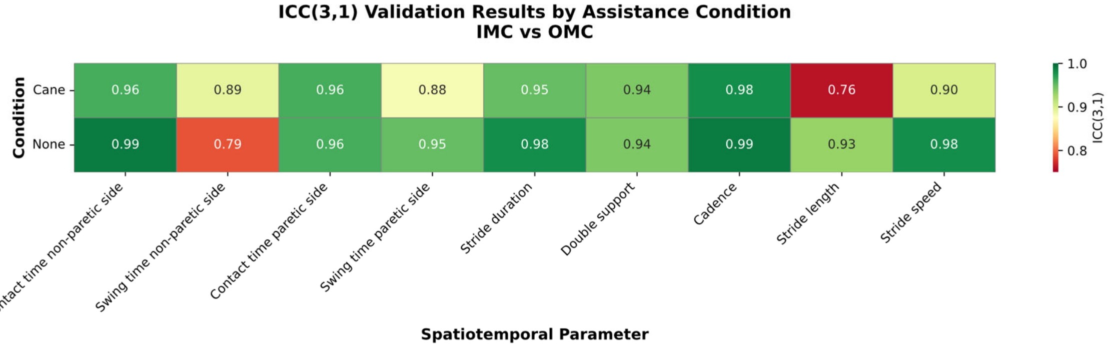

Supplementary Figure S2. Intraclass correlation coefficient (ICC(3,1)) heatmap for spatiotemporal parameters comparing IMC and OMC across assistive device groups (None, Cane). ICC values range from 0.75 (red) to 1.0 (green), with higher values indicating better agreement between systems. ICC(3,1) represents a two-way mixed effects model with absolute agreement.
